# Vergleich von Behandlungspfaden beim akuten Schlaganfall – eine qualitative multizentrische Studie in drei zuweisenden Kliniken eines Schlaganfallnetzwerkes

**DOI:** 10.1007/s00115-023-01453-z

**Published:** 2023-03-03

**Authors:** Franziska Herzog, Melek Sert, Johanna Hoffmann, Christina Stang, Fatih Seker, Jan Purrucker, Wolfgang Wick, Loraine Busetto, Christoph Gumbinger

**Affiliations:** 1 Neurologische Klinik, Universitätsklinikum Heidelberg, Heidelberg, Deutschland; 2 Abteilung für Neuroradiologie, Universitätsklinikum Heidelberg, Heidelberg, Deutschland; 3 Klinische Kooperationseinheit Neuroonkologie, Deutsches Krebsforschungszentrum, Heidelberg, Deutschland

**Keywords:** Endovaskuläre Thrombektomie, Intravenöse Thrombolyse, Teleneurologie, Akutversorgung, Versorgungsforschung, Endovascular thrombectomy, Intravenous thrombolysis, Teleneurology, Acute care, Health services research

## Abstract

**Hintergrund:**

In Schlaganfallnetzwerken verlegen Kliniken, die selbst keine endovaskuläre Thrombektomie (EVT) durchführen (hier: Primärkliniken), Patient*innen für diese Therapie in spezialisierte Schlaganfallzentren. Zur Verbesserung des Zugangs und des Managements der EVT muss der Fokus der Forschung nicht nur auf den spezialisierten Zentren, sondern auch auf den vorangehenden Prozessen in den Primärkliniken liegen.

**Fragestellung:**

Wie stellen sich die Schlaganfallbehandlungspfade in verschiedenen Primärkliniken dar und was wird in diesen Pfaden als Vor- und Nachteil gesehen?

**Methoden:**

Im Rahmen einer qualitativen multizentrischen Studie in drei Primärkliniken eines Schlaganfallnetzwerkes wurde die Versorgung von Schlaganfallpatient*innen mithilfe von nichtteilnehmenden Beobachtungen und 15 leitfadengestützten Interviews mit Beschäftigten unterschiedlicher Professionen erfasst und analysiert.

**Ergebnisse:**

Innerhalb der Schlaganfallbehandlungspfade wurden folgende Aspekte als vorteilhaft berichtet: (1) eine strukturierte und persönliche Ankündigung von Schlaganfallpatient*innen in der Primärklinik durch den Rettungsdienst, (2) ein effizienter Ablauf des Telekonsils, (3) die Notfallverlegung zur EVT durch die gleichen Rettungsdienstmitarbeitenden der Primärzuweisung und (4) die Integration von externen Neurolog*innen in klinikinterne Strukturen.

**Diskussion:**

Die Studie liefert einen Einblick in die z. T. unterschiedlichen Schlaganfallbehandlungspfade drei verschiedener Primärkliniken eines Schlaganfallnetzwerkes. Aus den Ergebnissen lassen sich Verbesserungspotenziale auch für andere Kliniken ableiten. Allerdings ist diese Studie zu klein, um verlässliche Aussagen über deren Wirksamkeit zu geben. Zukünftige Studien sollten daher untersuchen, ob Implementierungen der hier erarbeiteten Empfehlungen tatsächlich zu Verbesserungen führen bzw. unter welchen Bedingungen diese erfolgreich sind. Zur Sicherstellung der Patientenzentrierung sollte dabei auch die Perspektive von Betroffenen und Angehörigen miteinbezogen werden.

## Abstract (en)

**Background:**

In stroke networks, hospitals that do not provide thrombectomy (referring hospitals) refer patients to specialized hospitals (receiving hospitals) for this specific intervention. In order to improve the access and management of thrombectomy, the focus of research needs to be not only on the receiving hospitals but also on the prior stroke care pathways in referring hospitals.

**Objective:**

The purpose of this study was to investigate the stroke care pathways in different referring hospitals as well as the advantages and disadvantages associated with these pathways.

**Methods:**

A qualitative multicenter study was carried out in three referring hospitals of a stroke network. Stroke care was assessed and analyzed by using non-participant observations and 15 semi-structured interviews with employees in various health professions.

**Results:**

The following aspects were reported as advantageous within the stroke care pathways: (1) a structured and personal prenotification of the patient by the emergency medical service (EMS) members; (2) a more efficiently organized teleneurology workflow; (3) the provision of the secondary referral to thrombectomy by the same EMS members of the primary referral and (4) the integration of external neurologists into in-house structures.

**Conclusion:**

The study provides insights into different stroke care pathways of three different referring hospitals of a stroke network. The results can be used to derive potentials for improvement of other referring hospitals; however, this study is too small to provide reliable information about their potential effectiveness. Future studies should investigate whether implementation of these recommendations actually leads to improvements and under which conditions they are successful. To ensure patient-centeredness, the perspectives of patients and relatives should also be included.

Um eine flächendeckende Schlaganfallversorgung zu gewährleisten, haben sich in den letzten Jahren Kooperationen zwischen zur Thrombektomie verlegenden und zur Thrombektomie aufnehmenden Kliniken gebildet. Die hier beschriebenen Ergebnisse des Vergleichs der Schlaganfallbehandlung von drei verlegenden Kliniken eines Schlaganfallnetzwerkes können Kliniken mit ähnlichen Voraussetzungen dazu dienen, ihre eigenen Prozesse zu prüfen und ggf. zu optimieren.

Diese Studie basiert auf qualitativer Forschung, eine in der Neurologie recht neue Methode, die häufig auf (subjektiven) Interviews und Beobachtungen basiert und so Ansätze zur Prozessoptimierung liefern kann. Für eine Übersicht über die Anwendung und Bewertung qualitativer Forschung verweisen wir auf Busetto et al. [5].

## Hintergrund und Fragestellung

Sowohl der Einsatz der intravenösen Thrombolyse (IVT) als einzige medikamentöse Schlaganfallakuttherapie als auch der Einsatz der katheterbasierten endovaskulären Thrombektomie (EVT) sind zeitkritisch: Eine frühere Therapieeinleitung führt zu einem besseren Ergebnis [6, 7, 15]. Während die IVT an allen Kliniken mit Stroke-Unit verfügbar ist, kann die EVT aufgrund höherer Infrastruktur- und Personalanforderungen zurzeit nur an spezialisierten Kliniken erfolgen. Vor diesem Hintergrund haben sich netzwerkbasierte Kooperationen zwischen Kliniken mit und ohne Möglichkeit der EVT, u. a. durch koordinierte Verlegungen zur EVT sowie mittels teleneuro(radio)logischer Zusammenarbeit, als eine strukturierte Möglichkeit flächendeckender neurologischer Notfallbehandlung, etabliert [2].

Dies bedeutet, dass sich – je nach Patient*in – der Schlaganfallbehandlungspfad vom Symptombeginn über die Alarmierung des Rettungsdienstes, die Aufnahme in einer Klinik mit Möglichkeit zur IVT („Spoke“ im Sinne des sog. „Hub-and-Spoke-Modells“ in der Teleneurologie, hier: Primärklinik) und die Notfallverlegung zur EVT in ein Schlaganfallzentrum, bis hin zur Entlassung erstrecken kann [4]. Um ein besseres Verständnis für diesen Behandlungspfad zu entwickeln sowie um Ansatzpunkte für gezielte Verbesserungen aufzuzeigen, werden die Prozesse der einzelnen Abschnitte analysiert [1, 3]. Bisher lag der Fokus dieser Analysen vor allem auf den Schlaganfallzentren und weniger auf den diesen vorgeschalteten Primärkliniken [8]. Ebenfalls stehen häufig die Prozessketten und -zeiten im Mittelpunkt der Analyse, ohne diese in den jeweiligen Versorgungskontext der Kliniken einzubetten. Der Versorgungskontext liefert jedoch wichtige Hinweise darauf, in welchem Umfeld bestimmte Strategien funktionieren und welche Aspekte bei der Entwicklung von Qualitätsverbesserungsmaßnahmen beachtet werden sollten [5].

In der vorliegenden Arbeit werden detaillierte Behandlungspfade verschiedener (teleneurologisch unterstützten) Primärkliniken des Schlaganfallnetzwerkes „Schlaganfallkonsortium Rhein-Neckar (FAST)“ analysiert. Ziel ist es, die Vor- und Nachteile der jeweiligen Behandlungspfade innerhalb der Primärkliniken darzustellen. In diesem Zusammenhang kann der Vergleich verschiedener Kliniken sowohl zu einem besseren Verständnis der eigenen klinischen Praxis beitragen als auch zu einer fundierten Entscheidung, welche „funktionierenden“ Prozesse aus anderen Krankenhäusern in die eigene Klinik übertragen werden können.

## Studiendesign und Untersuchungsmethoden

Es erfolgte eine qualitative multizentrische Studie in drei verschiedenen Primärkliniken des FAST-Netzwerkes. Die Ergebnisse werden in Übereinstimmung mit den „Standards for Reporting Qualitative Research (SRQR)“ dargestellt [13].

### Sampling

Die Auswahl der Primärkliniken und der Interviewteilnehmenden erfolgte im Rahmen eines „purposive sampling“ mittels Kontaktpersonen des FAST-Netzwerkes. Um eine vergleichbare, aber möglichst heterogene Stichprobe zu erhalten, wurde darauf geachtet, dass die Primärkliniken regelmäßig mit einem Schlaganfallzentrum zusammenarbeiten, in unterschiedlichen Gebieten/Bundesländern des FAST-Netzwerkes lokalisiert sind und verschiedene Größen der Schlaganfallstationen aufweisen. Ergänzend hierzu wurden 15 leitfadengestützte Interviews geführt, in denen die Perspektiven von Beschäftigten unterschiedlicher Professionen, die an der akuten Schlaganfallversorgung beteiligt sind, erfasst werden sollten. Tab. 1 zeigt die Verteilung der interviewten Personen nach Funktion und Klinik.

**Table 1:** 

	Neurolog*in	Internist*in	Pflegekraft	Therapeut*in	RD-Mitarbeiter*in
Klinik A	1	2	2	1	–
Klinik B	1	1	1	1	1
Klinik C	1	2	1	–	–

*RD* Rettungsdienst

### Datenerhebung

Die Datenerhebung wurde von zwei Masterandinnen der Versorgungsforschung mit Erfahrung in qualitativer Forschung (FH, MS) durchgeführt, die nicht in die Patientenversorgung eingebunden waren. Zunächst wurden nichtteilnehmende Beobachtungen der akuten Versorgung von Schlaganfallpatient*innen – vom Eintreffen in der Notfallambulanz (NFA) bis zur stationären Behandlung oder Notfallverlegung – durchgeführt. Die Beobachtungen dauerten durchschnittlich 6,2 h (5–8 h) und fanden jeweils an 1 bis 3 Tagen zu unterschiedlichen Tageszeiten statt. Die beobachteten Ereignisse wurden aus unabhängigen Feldnotizen der beiden Forscherinnen zu Beobachtungsprotokollen konsolidiert. Der Interviewleitfaden für die zweite Teilerhebung enthielt offene Fragen zu den Erfahrungen der Beschäftigten im Kontext der Schlaganfallversorgung. Insgesamt dauerten die Interviews durchschnittlich 49,6 min (11,3–99,7 min). Sie wurden mit einem Audioaufnahmegerät digital aufgezeichnet und anschließend wortwörtlich transkribiert.

### Datenanalyse

Die Beobachtungsprotokolle und Interviewtranskripte wurden mithilfe der Datenanalysesoftware MAXQDA (2018, VERBI, Berlin) von jeweils zwei Forschenden unabhängig voneinander kodiert (FH, LB, MS). Mithilfe der Kodierungen erfolgte eine Analyse der Schlaganfallversorgung in den drei Kliniken. Für eine bessere Strukturierung der Ergebnisse wurde der Behandlungspfad in drei Phasen eingeteilt:

1. Prähospitale Phase: Abläufe vor der Ankunft der Schlaganfallpatient*innen in der NFA.
2. Akutmedizinische Phase: Behandlungsmaßnahmen vom Eintreffen der Patient*innen in der NFA bis zur stationären Aufnahme.
3. Stationäre Phase: Behandlungsmaßnahmen ab stationärer Aufnahme der Patient*innen bis zur Entlassung aus der Klinik.

### Ethikvotum

Die Studie wurde mit Zustimmung der zuständigen Ethikkommissionen (EK) durchgeführt (EK der Medizinischen Fakultät Heidelberg, S‑682/2017; EK der Landesärztekammer Baden-Württemberg, B‑F-2018-063 [Zweitvotum]; EK bei der Landesärztekammer Hessen, MC 145/2018 [Zweitvotum]). Alle interviewten Personen gaben ihr aufgeklärtes Einverständnis zur Studienteilnahme.

## Ergebnisse

**Figure 1:**
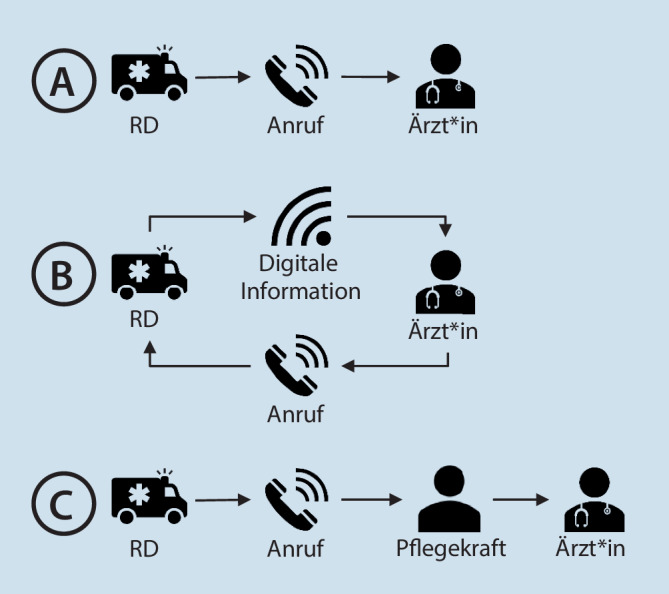

Die Ergebnisse werden entsprechend der Behandlungsphase dargestellt (Abb. 1, 2 und 3). Die größten Unterschiede und von den Beschäftigten berichteten Vor- und Nachteile bezogen sich auf die Ankündigung der Schlaganfallpatient*innen in der Primärklinik, den Ablauf der Teleneurologie, die Zusammenarbeit mit dem Rettungsdienst bei Verlegungen in das Schlaganfallzentrum und die Kooperation mit externen Neurolog*innen im Rahmen der stationären Behandlung.

**Figure 2:**
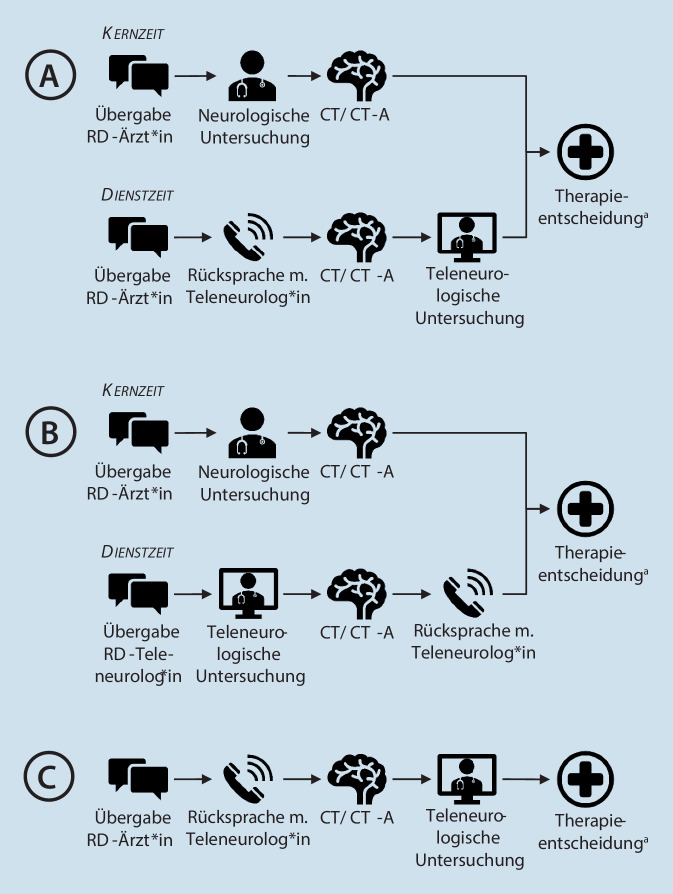

**Figure 3:**
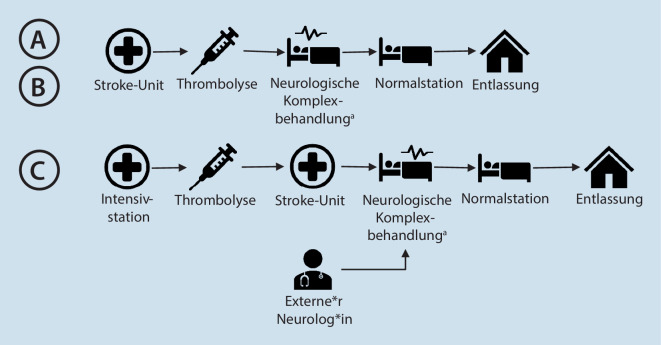

### Prähospitale Phase

In **Klinik A** werden Patient*innen mit Schlaganfallverdacht von den Rettungsdienstmitarbeitenden direkt bei den diensthabenden Ärzt*innen angemeldet. Nach (kurzer) telefonischer Übergabe kümmern sich die Ärzt*innen um die Freihaltung des Computertomographen (CT) und Sicherstellung eines Behandlungsplatzes auf der Stroke-Unit (SU).

Die Anmeldung von Schlaganfällen erfolgt in **Klinik B** über eine digitale Anzeigetafel in der NFA, die mit der Rettungsleitstelle verbunden ist. Auf dieser werden kurze Informationen zu den Patient*innen angezeigt (Alter, Geschlecht, Schlagwort und Ankunftszeit), bei Notfällen ertönt ein Alarmsignal. Die diensthabenden Ärzt*innen rufen daraufhin einen Rettungsdienstmitarbeitenden an und erfragen Auskünfte zu den Patient*innen. Anschließend werden die Neurolog*innen bzw. Teleneurolog*innen über die erwarte Symptomatik und Ankunftszeit der Patient*innen verständigt. Auch wird die Zeit bis zur Ankunft genutzt, um das CT freizuhalten und eine potenzielle IVT vorzubereiten.

In **Klinik C** werden Schlaganfallpatient*innen in der Regel bei den Pflegekräften der NFA angekündigt. Normalerweise kontaktieren diese anschließend die diensthabenden Ärzt*innen. In seltenen Fällen lassen sich die Rettungsdienstmitarbeitenden an die Ärzt*innen weiterleiten. Manchmal werden die Ärzt*innen auch erst bei Ankunft der Patient*innen benachrichtigt.

#### Vor- und Nachteile

Die Interviewpartner aus Klinik A und B sehen einen Vorteil in einer vorherigen Kommunikation zwischen Rettungsdienstmitarbeitenden und Ärzt*innen, weil dadurch eine bessere Vorbereitung und eine reibungslose akutmedizinische Behandlung erfolgen kann. Auch ein*e Ärzt*in der Klinik C wünscht sich eine vorherige Information durch den Rettungsdienst. Analog hierzu sieht ein*e Interviewpartner*in aus Klinik A die Kontaktierung des falschen Ansprechpartners als Nachteil, da es somit zu Zeitverzögerungen kommen kann:

„Also, Zeitverzögerung damit, dass der Neurologe einfach Fragen hat, die er wissen will. Und das einfach das Flüsterpost-Prinzip hat, wenn ich es erzählt bekomme. … Erzähle [ich dem Teleneurologen] die Geschichte [weiter], ist sie einfach anders, als es primär geschildert war“ (Klinik A, Internist*in, Interview 9).

Obwohl die Anmeldung durch die digitale Anzeigetafel in Klinik B von allen Befragten als Vorteil für eine schnellere Versorgung wahrgenommen wurde, wurde auch auf den Nachteil hingewiesen, dass es bei einer hohen Arbeitsbelastung in der Notaufnahme möglich ist, dass kein Beschäftigter die digitale Anzeigetafel beachtet und somit keine Vorbereitung auf Notfallpatient*innen erfolgen kann.

### Akutmedizinische Phase

In **Klinik A und B** gibt es jeweils zwei Versorgungspfade zu unterschiedlichen Arbeitszeiten. In der Kernzeit erfolgt die mündliche und schriftliche Übergabe des Rettungsdienstes an die Neurolog*innen. Diese führen anschließend eine kurze neurologische Untersuchung durch, stellen die medizinische Indikation für ein CT bzw. eine CT-Angiographie (CT-A). Nach der Bildgebung entscheiden die Neurolog*innen über die weitere Therapie. Im Fall einer IVT wird diese bereits in der Radiologie gestartet, anschließend werden die Patient*innen auf die SU und/oder in das Schlaganfallzentrum verlegt. In letzterem Fall nehmen die Ärzt*innen Kontakt zu den Neurolog*innen des Schlaganfallzentrums auf.

Wenn es bereits in der NFA klinisch wahrscheinlich erscheint, dass die Patient*innen zur EVT verlegt werden müssen, bleiben in **Klinik B** die Rettungsdienste vor Ort bis die Therapieentscheidung getroffen ist. Ein Transport erfolgt in diesen Fällen unmittelbar nach der Bildgebung und ggf. der bereits eingeleiteten IVT.

Außerhalb der Kernarbeitszeit kontaktieren in **Klinik A** die diensthabenden Ärzt*innen die Teleneurolog*innen. Diese entscheiden über eine Indikation zum CT bzw. CT‑A. Nach der Bildgebung erfolgt die teleneurologische Untersuchung in der NFA. Anschließend teilen die Teleneurolog*innen den Ärzt*innen die Therapieentscheidungen mit.

In **Klinik B** werden außerhalb der Kernarbeitszeit die Teleneurolog*innen von den diensthabenden Ärzt*innen telefonisch benachrichtigt, sobald die Patient*innen in der NFA angekommen sind. Die Teleneurolog*innen wählen sich in das Teleneurologiesystem des Krankenhauses ein und erhalten zunächst die mündliche Übergabe der Rettungsdienstmitarbeitenden. Nach der teleneurologischen Untersuchung stellen die Teleneurolog*innen die medizinische Indikation zur Bildgebung CT oder CT‑A. Im CT erfolgt eine Übergabe der Patient*innen von den Ärzt*innen der NFA an die Ärzt*inne der SU. Diese nehmen nach der Bildgebung Kontakt zu den Teleneurolog*innen auf und besprechen mit diesen die weitere Therapie, während die Patient*innen auf die SU verlegt werden.

In **Klinik C** findet die akutmedizinische Versorgung ausschließlich über die Teleneurologie statt. Bei Ankunft der Patient*innen in der NFA erfolgt zunächst die schriftliche und mündliche Übergabe der Rettungsdienstmitarbeitenden an die diensthabenden Ärzt*innen. Diese untersuchen anschließend die Patient*innen kurz und halten Rücksprache mit den Teleneurolog*innen über die CT- bzw. CT-A-Indikation. Nach der Bildgebung findet die teleneurologische Untersuchung und Therapieentscheidung in der NFA bzw. bei Verdacht zur IVT-Indikation auf der Intensivstation statt. Wenn eine Indikation zur IVT besteht, wird die Therapie auf der Intensivstation eingeleitet, ansonsten werden die Patient*innen auf die SU verlegt und dort überwacht.

#### Vor- und Nachteile

Die Mitarbeitenden in Klinik B berichteten, dass eine gute Kommunikation zwischen Neurolog*innen und Rettungsdienstmitarbeitenden in der Kernzeit zu einer zeitnahen Weiterverlegung der Patient*innen beitragen kann:

„… Wenn der Neurologe schon in der Notambulanz sieht, dass es ein schweres Mediasyndrom ist, also ein Klassiker eigentlich zum Weiterverlegen, dann sagen wir meistens dem Rettungsdienst, er soll schon mal zehn Minuten warten, Bild ist schnell gemacht, damit er dann am besten gleich weiterfahren kann“ (Klinik B, Neurolog*in, Interview 4).

Außerdem bestehen in Klinik B zwei Besonderheiten im Telekonsilablauf. Zum einen wird in dieser Klinik die teleneurologische vor der CT-Untersuchung durchgeführt. Nach Aussage eines Rettungsdienstmitarbeitenden habe dies den Vorteil, dass er*sie Informationen zu den Patient*innen direkt an die Teleneurolog*innen übergeben und mögliche Rückfragen beantworten könne.

Zum anderen beschrieben die Mitarbeitenden aus Klinik B die effektive Arbeitsteilung zwischen NFA- und SU-Ärzt*innen. Während die NFA-Ärzt*innen für die Behandlung der Patient*innen und Kommunikation mit den Teleneurolog*innen bis zur CT-Diagnostik verantwortlich sind, übernehmen anschließend die SU-Ärzt*innen diese Aufgaben. Ein Vorteil wurde von einem*r Neurolog*in dieser Klinik darin gesehen, dass das Team der NFA entlastet und die Patient*innen besser versorgt werden.

### Stationäre Phase

In allen drei Kliniken finden auf der SU das Monitoring, die pflegerische Versorgung sowie verschiedene Untersuchungen und Therapien (Ergotherapie, Logopädie, und Physiotherapie) statt. In **Klinik A und B** gibt es jeden Tag eine interdisziplinäre Teambesprechung und eine ärztliche Visite mit den Neurolog*innen. In **Klinik C** erfolgen neurologische Konsile durch die Neurolog*innen eines medizinischen Versorgungszentrums (MVZ). Diese Neurolog*innen begleiten die Ärzt*innen jeden Tag bei der ärztlichen Visite auf der SU. Nach der neurologischen Komplexbehandlung auf der SU werden die Patient*innen aller drei Kliniken auf eine Normalstation verlegt und anschließend nach Hause oder in eine Rehabilitationsklinik entlassen.

#### Vor- und Nachteile

Von den Mitarbeitern in Klinik C wurde thematisiert, dass die Kooperation mit externen Neurolog*innen vorteilhaft bei der Sicherstellung der fachärztlichen Behandlung in kleineren Kliniken ohne eigene neurologische Fachabteilung ist. Der*die behandelnde Assistenzärzt*in und der*die Neurolog*in des MVZ nannten als Nachteil der Zusammenarbeit, dass die Visiten ohne den*die zuständigen (internistischen) Oberärzt*in ablaufen. Außerdem wurde der bisherige papierbasierte Informationsaustausch als veraltet und verbesserungswürdig beschrieben.

## Diskussion

Die Hauptunterschiede sowie Vor- und Nachteile der Behandlungspfade in den drei Primärkliniken zeigten sich in der prähospitalen Phase bei der Ankündigung von Schlaganfallpatient*innen, in der akutmedizinischen Phase beim Ablauf der Teleneurologie und der Zusammenarbeit mit dem Rettungsdienst zur Verlegungen in das Schlaganfallzentrum sowie in der stationären Phase bei der Kooperation mit externen Neurolog*innen.

### Ankündigung der Schlaganfallpatient*innen:

Die Information erfolgte digital oder telefonisch und die Ansprechpartner waren Ärzt*innen oder Pflegekräfte. Als vorteilhaft wurden dabei eine direkte Kontaktierung des richtigen ärztlichen Ansprechpartners und ein persönliches Gespräch zwischen Ärzt*innen und Rettungsdienstmitarbeitenden gesehen. Analog hierzu kamen Ragoschke-Schumm et al. [14] in ihrer Übersichtsarbeit zum prähospitalen Schlaganfallmanagement zu dem Ergebnis, dass eine vorherige Ankündigung von Schlaganfallpatient*innen bei dem Team der NFA durch die Rettungsdienstmitarbeitenden einer der wichtigsten zeitsparenden Faktoren bei der Akutversorgung ist. Die Autor*innen sehen außerdem einen Gewinn in der Nutzung digitaler Assistenzsysteme für den strukturierten Transfer der Patientendaten zwischen Rettungsdienst und Krankenhaus. Auch in der vorliegenden Studie wurden digitale Assistenzsysteme als Vorteil für eine strukturierte präklinische Versorgung gesehen. Allerdings zeigten die Ergebnisse unserer Studie auch, dass die akustischen Alarmierungen bei hoher Arbeitsbelastung in der NFA manchmal nicht wahrgenommen werden.

Es erscheint daher empfehlenswert, dass Primärkliniken Prozessänderungen erwägen, die eine strukturiertere prähospitale Ankündigung und ein persönliches Gespräch zwischen Rettungsdienstmitarbeitenden und zuständigen Ärzt*innen ermöglichen. Dies könnte durch gemeinsame Fallbesprechungen oder Fortbildungen unterstützt werden [10]. Zusätzlich sollte bei Implementierung neuer (digitaler) Systeme berücksichtigt werden, dass diese ausreichend in den Klinikalltag integriert werden, damit sie nicht disruptiv sind.

### Ablauf der Teleneurologie:

Alle Kliniken stellten die neurologische Versorgung der Patient*innen außerhalb der Kernzeit oder zu jedem Zeitpunkt durch eine teleneurologische Kooperation mit dem Schlaganfallzentrum sicher. Während es in zwei Kliniken nur eine*n behandelnden Ärzt*in vor Ort gab, wurde in einer Klinik die Verantwortlichkeit zwischen den Ärzt*innen der NFA und der SU aufgeteilt. Dies wurde von den Mitarbeitenden als Vorteil wahrgenommen. Andere Primärkliniken können überprüfen, ob eine solche Arbeitsteilung auch in ihren Kliniken möglich ist und ob dies einen positiven Einfluss auf die Arbeitsbelastung der Mitarbeitenden hat.

In unserer Studie zeigten sich auch Unterschiede in dem Behandlungspfad der Teleneurologie. Während in zwei Kliniken das CT vor der teleneurologischen Untersuchung durchgeführt wurde, erfolgte in einer Klinik die Teleneurologie vor dem CT. Die letztere Vorgehensweise hat den Vorteil, dass ein Austausch zwischen Rettungsdienstmitarbeitenden und Teleneurolog*innen stattfinden kann. Ein vergleichbares Ergebnis wurde in einer Registerstudie mit 1020 Patient*innen in einem Teleneurologienetzwerk in den USA berichtet. Die Autor*innen kamen außerdem zu dem Ergebnis, dass ein CT vor der Teleneurologie zu einer längeren Zeit bis zur Kontaktierung der Teleneurolog*innen sowie einer längeren Zeit bis zur Verabreichung der IVT (DTNT, „door-to-needle-time“) führt [9].

Ein möglicher Grund, warum Primärkliniken zuerst das CT vor der Teleneurologie durchführen möchten, obwohl o. g. Auswertung einen medizinischen Vorteil der anderen Abfolge suggeriert, könnte in der angestrebten Einhaltung des Qualitätssicherungsparameters „Durchführung der Bildgebung innerhalb 30 min“ liegen (Grenze 30 min nach Aufnahme).

Vor diesen Hintergrund sollte bei der Festlegung der Qualitätssicherungsparameter untersucht werden, ob es sinnvoll ist, diesen Parameter auch bei Teleneurologiepatient*innen zu erheben bzw. Ausnahmen für diese Patient*innen zu definieren. In den betreffenden Primärkliniken sollte überprüft werden, ob eine Änderung des Ablaufes der Teleneurologie zu einer verbesserten DTNT führen könnte oder ob historisch gewachsene bzw. kontextspezifische Gründe gegen eine Veränderung bestehen. Durch eine strukturierte Analyse von Barrieren und Förderfaktoren der Teleneurologie aus Patient*innen- und Mitarbeitendensicht, könnten zukünftige Studien zu einem besseren Verständnis und gezielteren Handlungsempfehlungen für diesen wichtigen Behandlungsaspekt beitragen.

### Verlegung zum Schlaganfallzentrum:

Eine der drei Kliniken der vorliegenden Studie beschrieb, dass bei Verdacht auf Weiterverlegung zur EVT die Neurolog*innen die Rettungsmitarbeitenden über diese Vermutung informieren. Dies hat den Vorteil, dass die Rettungsmitarbeitenden in der NFA die Primärdiagnostik abwarten und anschließend ohne Zeitverzögerung den Transport in das Schlaganfallzentrum durchführen können. Eine retrospektive Studie in Australien stellte fest, dass die Zeit bis zur Aufnahme im Schlaganfallzentrum bei Patient*innen, die von dem gleichen Rettungsdienstteam verlegt wurden, signifikant verkürzt war. Wenn die Rettungsdienstteams in der Primärklinik warteten, konnten zusätzliche zeitliche Vorteile festgestellt werden [12]. Dieses Ergebnis sollte auch im deutschen Versorgungskontext überprüft werden und bei Bestätigung zu einer Empfehlung dieser Verfahrensweise führen.

### Kooperation mit externen Neurolog*innen:

Während in zwei Kliniken Neurolog*innen zu den Kernarbeitszeiten vor Ort waren, wurden die Neurolog*innen in einer Klinik über ein MVZ zu den täglichen Visiten und neurologischen Konsilen hinzugezogen. Ein Gutachten des Zentralinstituts für die kassenärztliche Versorgung in der Bundesrepublik Deutschland zur Zusammenarbeit in der ambulanten und stationären Versorgung beschrieb die Integration ambulanter Fachärzt*innen in Klinikbereiche als Lösungsansatz, um stationäre Versorgungslücken zu schließen [11]. In der vorliegenden Studie wurden jedoch auch Probleme bei der Zusammenarbeit zwischen dem externen und internen Klinikpersonal beschrieben. Diese Herausforderungen könnten durch eine engere Integration von MVZs in die internen Abläufe der Primärkliniken, z. B. durch die Durchführung gemeinsamer Besprechungen oder die gemeinsame Nutzung elektronischer Dokumentation adressiert werden.

### Stärken und Limitationen der Studie

Durch den Einsatz von Beobachtungen und Interviews konnten wir Behandlungspfade der akuten Schlaganfallversorgung sowie deren Vor- und Nachteile aus Mitarbeitendensicht identifizieren. Die Triangulierung der beiden Methoden und die Einbeziehung verschiedener Professionen hatte den Vorteil, dass wir ein besseres Verständnis für die Abläufe gewinnen und diese Erkenntnisse mit der individuellen Sicht unterschiedlicher interviewter Personen vergleichen konnten. Eine weitere Stärke der Studie besteht in der Fokussierung auf „kleinere“ Kliniken außerhalb urbaner Gebiete, die normalerweise eher selten im Zentrum der Forschung liegen. Da diese jedoch häufig für die Primärversorgung von Schlaganfallpatient*innen verantwortlich sind, kann eine Identifikation von Verbesserungspotenzialen in diesen Kliniken einen großen Einfluss auf eine flächendeckende Schlaganfallversorgung haben.

Als Limitation der Studie ist u. a. zu beachten, dass durch die begrenzten Präsenzzeiten der Forschenden in den Primärkliniken der Einschluss der Interviewpartner weniger gut gesteuert werden konnte. Dies hatte zur Folge, dass keine gleichmäßige Repräsentation der Professionen zwischen den Kliniken erreicht werden konnte, z. B. wurde nur in einer Klinik ein*e Rettungsdienstmitarbeiter*in rekrutiert. Zusätzlich ist zu beachten, dass in dieser Arbeit nur wenige Primärkliniken eines einzigen Schlaganfallnetzwerkes betrachtet wurden. Eine Übertragung der Ergebnisse auf Kliniken mit anderen Kontextfaktoren und innerhalb anderer Schlaganfallnetzwerke muss geprüft werden. Außerdem weisen wir darauf hin, dass die subjektive Einschätzung einzelner Personen nicht einer evidenzbasierten Empfehlung gleichzusetzten ist. Ebenfalls war die Überprüfung der individuellen Aussagen auf ihre objektive Richtigkeit nicht Ziel dieser Arbeit.

## Schlussfolgerung

Insgesamt lieferte unsere Studie einen Einblick in die Schlaganfallbehandlungspfade verschiedener Primärkliniken eines Schlaganfallnetzwerkes. Aus den berichteten Vor- und Nachteilen unterschiedlicher Prozesse wurden unter Einbeziehung der Literatur Verbesserungspotenziale herausgearbeitet. Allerdings ist jeweils im Einzelfall zu prüfen, ob diese Empfehlungen sinnvoll in den Behandlungsablauf anderer Kliniken integriert werden können oder ob eigene Lösungen die regionalen Besonderheiten wirksamer adressieren. Zukünftige Studien sollten daher die Ergebnisse dieser Studie in einer größeren Krankenhausstichprobe überprüfen und untersuchen, ob Implementierungen der hier erarbeiteten Empfehlungen tatsächlich zu Verbesserungen führen bzw. unter welchen Bedingungen diese erfolgreich sind. Zur Sicherstellung der Patientenzentrierung sollte dabei auch die Perspektive von Betroffenen und Angehörigen miteinbezogen werden.

## Fazit für die Praxis

- Prähospital werden strukturierte Ankündigungen und Gespräche zwischen Ärzt*innen und Rettungsdienstmitarbeitenden als Gewinn für eine bessere Vorbereitung der Akutsituation gesehen.
- Die Durchführung des Telekonsils vor der CT kann zu einer stärkeren Einbeziehung des Rettungsdienstes führen und ist, ebenso wie Absprachen zwischen den Ärzt*innen, eine Möglichkeit der Effizienzsteigerung des Telekonsilablaufes. Zu diskutieren ist hier der potenziell kontraproduktive Qualitätssicherungsparameter für die telemedizinische Schlaganfallversorgung „Bildgebung innerhalb 30 min“.
- Durch Warten des Rettungsdienstes in der Primärklinik bei klinischem Verdacht der Weiterverlegung zur EVT würden Zeitverzögerungen beim Transport vermieden.
- Kooperationen mit externen Neurolog*innen in der stationären Behandlung stellen eine fachärztliche Behandlung sicher, es können jedoch strukturelle oder kontextbezogene Probleme auftreten. Diese sollten z. B. durch einen Informationsaustausch zwischen externen und Klinikpersonal adressiert werden.
